# Octa evaluation of macular and optic disc vascular changes after PRP

**DOI:** 10.1007/s00417-026-07220-5

**Published:** 2026-04-01

**Authors:** Deniz Dilan Karabacak, Suzan Doğruya, Muhammed Altınışık, Özcan Rasim Kayıkçıoğlu

**Affiliations:** 1 Ophthalmology, Bornova Türkan Özilhan State Hospital, İzmir, Turkey; 2 Department of Ophthalmology, Uşak University, Uşak, Turkey; 3 Department of Ophthalmology, Manisa Celal Bayar University, Manisa, Turkey; 4 Ophthalmology, Private Practice, Manisa, Turkey

**Keywords:** Diabetic retinopathy, Optical coherence tomography angiography, Panretinal photocoagulation, Vascular density

## Abstract

**Purpose:**

This study aimed to evaluate the impact of panretinal photocoagulation (PRP) on macular and optic nerve head microvascular parameters using optical coherence tomography angiography (OCTA) in eyes with proliferative diabetic retinopathy (PDR) and very severe non-proliferative diabetic retinopathy (NPDR).

**Material and Method:**

In this prospective study, 56 treatment-naive eyes diagnosed with PDR or very severe NPDR and without diabetic macular edema were enrolled. PRP was performed over two sessions using a pattern scan laser in multispot mode. For each eye, the total laser ablation area (LAA) was calculated. OCTA imaging was performed before PRP, after the first PRP session, and at the 1-month follow-up. The following parameters were assessed: central macular thickness (CMT), subfoveal choroidal thickness (SFCT), foveal avascular zone (FAZ), superficial capillary plexus (SCP) vascular density (VD), deep capillary plexus (DCP) VD, and radial peripapillary capillary plexus (RPCP) VD.

**Results:**

Twenty-seven eyes had very severe NPDR and 29 had PDR. One month after PRP, CMT increased significantly, while SFCT and best-corrected visual acuity remained unchanged. SCP-VD values (whole image, superior/inferior hemi-fields, parafoveal and perifoveal regions) were significantly reduced both 30 min and 1 month after PRP. DCP-VD values were significantly reduced at 30 min in all regions but returned to baseline by month one. FAZ area and perimeter did not change significantly, whereas foveal density-300 (FD-300) decreased both at 30 min and at one month. Regarding optic nerve head parameters, RPCP-VD inside the disc decreased at 30 min, and peripapillary RPCP-VD decreased significantly at the one-month visit. No correlation was found between LAA and changes in any OCTA parameter or OCT metrics.

**Conclusion:**

OCTA revealed that PRP induces measurable microvascular alterations in both the macula and optic nerve head. These findings suggest that PRP has acute and subacute effects on retinal and peripapillary microvasculature independent of total laser ablation area.

## Key Message

What is known.

• Panretinal photocoagulation (PRP) is the standard treatment for proliferative diabetic retinopathy (PDR) and may alter retinal vascular structure.

• Optical coherence tomography angiography (OCTA) is a non-invasive imaging modality that enables quantitative evaluation of retinal and optic disc microvasculature.

What is new.

• This study quantitatively assessed early and one-month vascular changes in the macula and optic disc following PRP using OCTA.

• Significant reductions in vessel density were observed early after PRP in both superficial (SCP) and deep (DCP) capillary plexuses; while DCP values returned to baseline by one month, some SCP parameters remained reduced.

• Peripapillary vessel density (RPCP) showed significant early and sustained decreases in arterio-venous analysis post-PRP, but these changes were not correlated with the total laser application area.

## Introduction

Diabetic retinopathy (DRP) remains the leading cause of visual impairment among working-age adults worldwide. The global prevalence of DR among adults aged 40 years and older with diabetes is estimated at 34.6% (93 million individuals), while sight-threatening DRP affects approximately 10.2% (28 million individuals) [1]. 

Panretinal photocoagulation (PRP) is a well-established treatment modality with proven efficacy in altering the natural course of proliferative diabetic retinopathy (PDR). Large randomized clinical trials have demonstrated that PRP substantially reduces the risk of severe vision loss. Its primary mechanism is thought to involve decreasing ischemia-driven vascular endothelial growth factor (VEGF) production by ablating metabolically active photoreceptors and thereby reducing the hypoxic stimulus. Consequently, PRP promotes regression of retinal neovascularization and limits complications that may lead to irreversible visual decline. Nevertheless, PRP may also induce both transient and long-term alterations in retinal structure and vasculature, in part through inflammatory pathways [2, 3]. 

Optical coherence tomography angiography (OCTA) has emerged as an innovative, non-invasive imaging technology capable of visualizing retinal and choroidal microvasculature in three dimensions. Its ability to delineate the superficial and deep capillary plexuses provides valuable insight into the pathophysiology and progression of vascular diseases, including DRP [4]. With the increasing availability of OCTA, it has become feasible to assess PRP-related changes within individual vascular plexuses.

However, only a limited number of studies have evaluated the effects of PRP on retinal microvasculature using OCTA, and the available findings are inconsistent. Some OCTA studies suggest that PRP may alter macular perfusion and/or FAZ-related metrics, whereas others report little or no measurable microvascular change, likely reflecting differences in study design, laser parameters, and timing of post-treatment imaging [5–10]. Given these discrepancies, additional research is required to clarify early and intermediate vascular responses following PRP.

To better understand the temporal profile of PRP-induced vascular changes, it is essential to distinguish between the immediate hemodynamic response and subsequent subacute vascular adaptation. Laser photocoagulation produces controlled thermal injury in the peripheral retina and is known to induce acute inflammatory and autoregulatory vascular responses, including transient vasoconstriction and cytokine release, which may lead to short-term perfusion alterations shortly after treatment [3, 11]. These early changes may be detectable within minutes of laser application. In contrast, vascular remodeling, redistribution of retinal blood flow, and potential reperfusion processes are more likely to evolve over weeks. Although several OCTA studies have evaluated retinal vascular parameters at one month or later following PRP [6, 12], ultra-early microvascular alterations have rarely been investigated.

Therefore, in the present study, OCTA parameters were specifically assessed at 30 min to capture acute vascular responses and at one month to evaluate early stabilization or recovery. In addition to its biological relevance, the 30-minute time point was selected as a methodologically appropriate interval, allowing sufficient time for patients to recover from the laser procedure, achieve stable fixation, and cooperate reliably with OCTA imaging, while still reflecting the acute post-treatment phase. We hypothesized that PRP would induce an immediate reduction in retinal vessel density followed by partial normalization during the subacute phase.

In this study, we aimed to investigate PRP-related alterations in macular and optic nerve head microvascular parameters using OCTA in eyes with high-risk DRP, focusing on both acute (30-minute) and subacute (one-month) effects.

## Materials and methods

Ethics committee approval for this study was obtained from the university ethics committee (22.03.2021/ 157), and the study was conducted in accordance with the principles of the Declaration of Helsinki, with informed consent obtained from all patients.

This study included 56 eyes of 56 patients who presented to the ophthalmology clinic of a tertiary university hospital between June 2019 and March 2021 and were diagnosed with DRP.

Inclusion criteria were diagnosis of type 2 diabetes mellitus (DM), presence of proliferative diabetic retinopathy (PDR) or very severe non-proliferative diabetic retinopathy (NPDR) according to the International Diabetic Retinopathy Severity Scale, evidence of peripheral ischemia on fundus fluorescein angiography (FFA), spherical equivalent between − 3.0 and + 3.0 diopters, intraocular pressure (IOP) below 21 mmHg, treatment-naive status, and successful completion of laser treatment. For patients with bilateral diabetic retinopathy, the eye scheduled for PRP treatment during the study period was included. If both eyes required treatment, the eye treated first was selected to avoid inter-eye correlation bias.

Exclusion criteria included media opacities impairing image quality (e.g., cataract, corneal opacity, intravitreal hemorrhage), ocular diseases other than DRP, diabetic macular edema (DME) which could affect visual acuity, history of eye surgery other than uncomplicated cataract surgery, or any eye surgery within the last six months. Patients with severe disc neovascularization (NVD) or tractional fibrovascular components interfering with optical coherence tomography angiography (OCTA) imaging in the macula or optic disc were also excluded. OCTA images of quality below Q5, or those with blinking/motion artifacts or uncorrectable segmentation errors, were excluded. Additionally, 14 patients were lost to follow-up, whose data was not included.

Demographic characteristics, duration of DM, HbA1c values (%), and presence of systemic comorbidities were recorded. Best corrected visual acuity (BCVA) was measured using the Snellen chart, converted to decimal values, and then to LogMAR before and one month after laser treatment. IOP was measured with non-contact tonometer (Canon Full Auto Tonometer TX-F^®^, Canon, USA), refractive values with an autorefractometer (Canon RK-F1, Tokyo, Japan), and lens status (phakic or pseudophakic) was evaluated. Pupillary dilation and fundus examinations were performed with a 90 D non-contact lens, and optical coherence tomography (OCT) was conducted. Cases with central macular thickness (CMT) above 250 μm or intraretinal/subretinal fluid were considered DME and excluded. FFA was performed in patients with PDR and very severe NPDR, and those with peripheral ischemia were selected.

### PRP laser parameters

Pupil dilation was achieved using 1% tropicamide and 2.5% phenylephrine, and topical anesthesia was applied with proparacaine drops. All patients were treated by an experienced ophthalmologist using the PASCAL^®^ laser device (Topcon Medical Laser Systems, Santa Clara, CA, USA), a 532 nm frequency-doubled Nd: YAG laser, in multi-scan mode (2 × 2 and 3 × 3) with a Mainster 165 PRP lens (magnification factor 1.96; Ocular Instruments Inc., Bellevue, WA, USA).

Laser parameters were: power 800–2000 mW, 200 μm spot diameter, and 20 ms pulse duration, creating a light yellow-white burn on the retina. Spot spacing was set at 0.75 times the spot diameter. Using the device’s multi-pattern scanning feature, 2 × 2 and 3 × 3 pattern laser treatments were applied. PRP was delivered from the peripheral arches to the ora serrata, maintaining a two-disc diameter distance from the fovea temporalis and optic disc, in two sessions spaced one week apart.

Although the mean laser power appeared higher than values reported in the DRS and ETDRS, this is attributable to the use of short pulse duration (20 ms) and pattern scan laser delivery. Shorter pulse durations require higher power settings to achieve a clinically equivalent light yellow-white burn while delivering lower total thermal energy to the retina. This approach has been shown to reduce collateral retinal damage and patient discomfort compared to conventional long-pulse photocoagulation.

OCTA images of 6 × 6 mm macula and 4.5 × 4.5 mm optic disc were acquired before PRP. To assess acute PRP effects, OCTA measurements were repeated 30 min after the first laser session. One week later, the second PRP session was performed, and OCTA scans were repeated at one month post-treatment. Imaging was performed by the same experienced technician, and images with quality below Q5 were repeated.

Since the 200 μm spot diameter on the device corresponds to 392 μm on the retina (200 × 1.96), the ablation area of each laser shot was calculated as 0.1207 mm² (πr²). Total laser ablation area (LAA) per eye was calculated, and correlations between OCTA parameter changes at one month and total LAA post-PRP were evaluated.

### OCTA measurement parameters

OCTA was performed using the Avanti RTVue XR AngioVue (version 2018.0.0.14; Optovue, Inc., Fremont, CA, USA), with an 840 nm light source, infrared video eye-tracking system, and motion correction technology, acquiring 70,000 A-scans/sec.

Macular 6 × 6 mm OCTA images were divided into concentric rings of 1, 3, and 6 mm for foveal, parafoveal, and perifoveal regions. Superficial capillary plexus vascular density (SCP-VD) and deep capillary plexus vascular density (DCP-VD) were measured in the whole image, superior/inferior hemispheres, fovea, parafovea, and perifovea. Foveal avascular zone (FAZ) area (FAZ-A), FAZ perimeter (PERIM), foveal vascular density (FD), and FD-300 (vascular density in a 300 μm ring around FAZ) were automatically calculated.

Subfoveal choroidal thickness (SFCT) was measured manually using the “Enhanced HD Line” module, from the outer border of the hyperreflective RPE line to the choroidoscleral junction. Radial peripapillary capillary plexus vascular density (RPCP-VD) was evaluated in 4.5 × 4.5 mm optic disc OCTA images in patients without NVD, measuring small vessels (SV) and all vessels (AV) in both the inside-disc and peripapillary regions.

**Fig. 1:**
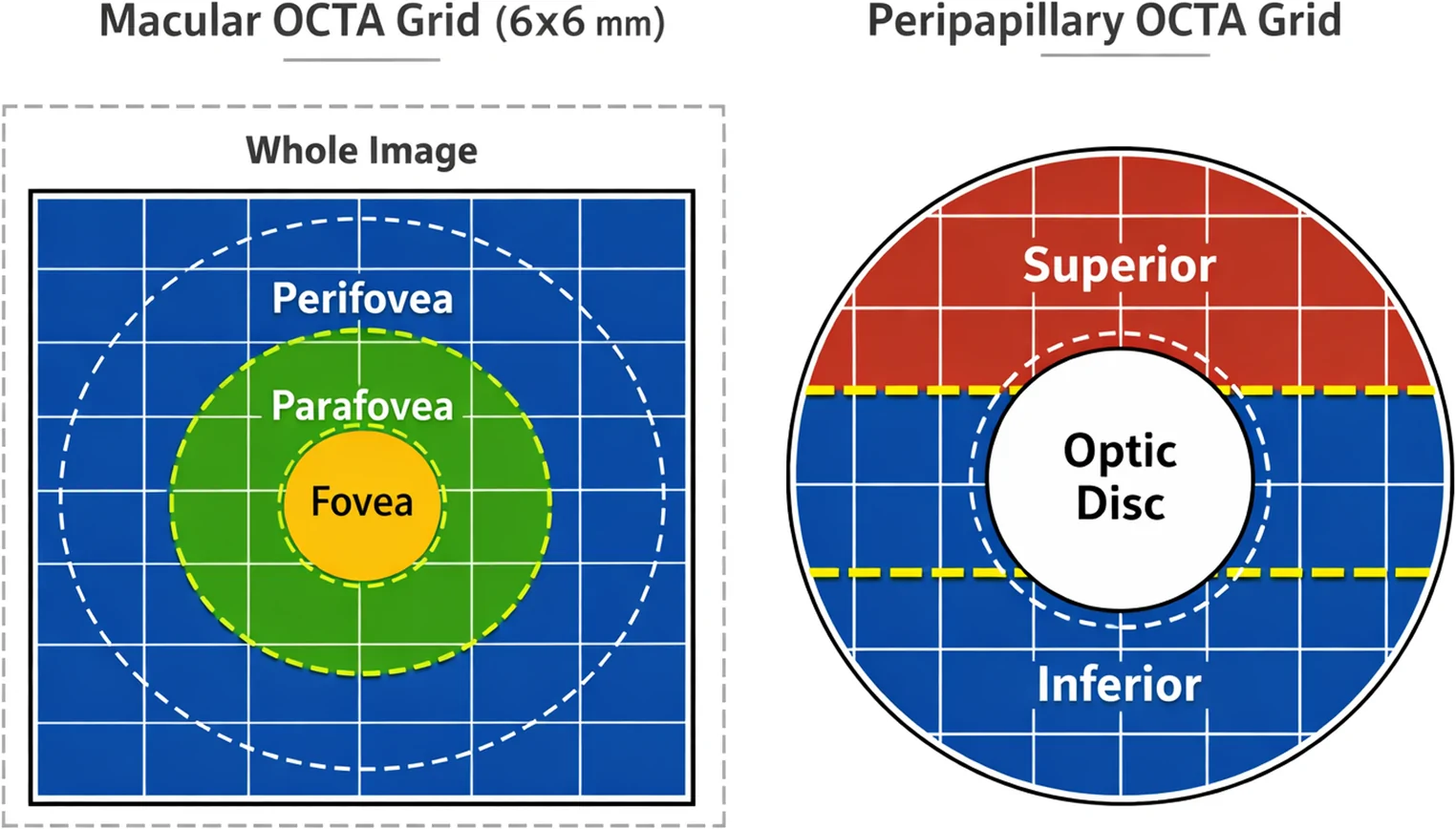
Schematic illustration of OCTA measurement grids. The macular OCTA scan represents a 6 × 6 mm area, subdivided into the fovea (central 1 mm), parafovea (1–3 mm ring), and perifovea (3–6 mm ring) regions, as well as whole-image analysis. The peripapillary OCTA scan illustrates the 4.5 × 4.5 mm optic disc area, including inside-disc and peripapillary regions, with superior and inferior hemispheric division used for vascular density analysis

A schematic illustration of the OCTA measurement grids, including whole-image analysis and superior–inferior hemispheric division, is provided in Fig. 1.

### Statistical analysis

Statistical analyses were performed using SPSS 24 (SPSS Inc., Chicago, IL, USA). Numerical data are presented as mean ± standard deviation; categorical data as number (%). Normality of numerical variables was tested with the Kolmogorov–Smirnov test (*P* > 0.05 considered parametric).

Repeated measures ANOVA was used for normally distributed repeated data. If Mauchly’s sphericity test indicated violation (*P* < 0.05), Greenhouse–Geisser correction was applied. Significant differences were further analyzed with Bonferroni-corrected post hoc tests. Nonparametric repeated measures were evaluated with the Friedman test, followed by Wilcoxon signed-rank test with Bonferroni correction for pairwise comparisons.

Correlations between LAA and OCTA/OCT parameters were assessed using Spearman’s test. Correlation coefficients (r) were interpreted as weak (< 0.3), moderate (0.3–0.7), or strong (> 0.7). All analyses used a 95% confidence interval, and *P* < 0.05 was considered statistically significant.

## Results

Fifty-six eyes from 56 patients diagnosed with PDR or very severe NPDR and scheduled for PRP were included in the study. The mean age of the cohort was 56.39 ± 8.92 years, comprising 37 men (66.1%) and 19 women (33.9%). All patients had type 2 DM with a mean disease duration of 11.98 ± 7.07 years and an average HbA1c of 10.30 ± 2.02%. When stratified by DRP stage, 29 eyes (51.8%) had PDR, and 27 eyes (48.2%) had very severe NPDR (Table 1). Among the fellow eyes of patients in the very severe NPDR group, high-risk PDR was present in 18 patients (66.6%), and limited peripheral tractional retinal detachment was observed in 4 patients (14.8%). Early laser intervention in this group was determined based on fellow eye status and socioeconomic considerations.

**Table 1:** Baseline characteristics of the study population

Characteristic	Number (*n*)	Percentage (%)
Systemic Comorbidities		
Not reported	21	43.75
Hypertension (HT)	20	35.7
Hypercholesterolemia	3	6.25
Coronary artery disease (CAD)	3	6.25
HT + CAD	4	8.33
Diabetic Retinopathy Stage		
Very severe NPDR	27	48.2
PDR – NVE only	18	32.1
PDR – NVD only	5	8.9
PDR – NVE + NVD	6	10.7

*Abbreviations*: *HT* Hypertension, *CAD* Coronary artery disease, *DRP* Diabetic retinopathy, *NPDR* Non-proliferative diabetic retinopathy, *PDR* Proliferative diabetic retinopathy, *NVE* Neovascularization elsewhere, *NVD* Neovascularization of the disc

Ophthalmologic examination revealed a mean BCVA of 0.30 ± 0.28 logMAR, IOP of 15.95 ± 3.27 mmHg, and spherical equivalent of 1.25 ± 0.50 D. Forty-six eyes (82.1%) were phakic, and 10 eyes (17.9%) were pseudophakic. One month following PRP, BCVA was 0.30 ± 0.12 logMAR and IOP was 15.75 ± 2.21 mmHg, with no significant differences compared to baseline (*p* > 0.05).

Macular and optic disc vascular parameters (in eyes without NVD) assessed by OCTA before the first PRP session, 30 min post-PRP, and at the one-month follow-up are presented in Table 2.

**Table 2:** Macular and optic disc OCTA measurements before PRP, 30 min after PRP, and at the 1-month follow-up, with corresponding *P*-values

Parameter Name	Before PRP	30 min after PRP	1 month after PRP	*P*-value (p*, p1, p2, p3)
CMT (Central Macular Thickness, µm)	298.07 ± 83.86	298.43 ± 84.56	313.09 ± 93.78	< 0.0001 p1 > 0.05 p2 = 0.0005 p3 = 0.0006
SFCT (Subfoveal Choroidal Thickness, µm)	254.16 ± 44.47	258.63 ± 45.68	261.07 ± 44.81	0.084
SCP-VD Whole Image (%)	46.66 ± 4.52	44.94 ± 4.44	45.29 ± 3.97	0.002 p1 = 0.002 p2 = 0.010 p3 = 1
SCP-VD Superior Hemi (%)	46.65 ± 4.58	44.71 ± 4.61	45.10 ± 4.21	0.012 p1 = 0.006 p2 = 0.020 p3 > 0.05
SCP-VD Inferior Hemi (%)	46.63 ± 4.67	45.20 ± 4.52	45.49 ± 3.97	0.01 p1 = 0.012 p2 = 0.026 p3 = 1
SCP-VD Fovea (%)	20.33 ± 6.52	19.82 ± 7.52	20.97 ± 6.75	0.319
SCP-VD Parafovea (%)	46.65 ± 5.15	44.14 ± 4.84	44.55 ± 5.18	0.007 p1 = 0.0006 p2 = 0.0007 p3 = 1
SCP-VD Perifovea (%)	47.45 ± 4.77	45.76 ± 4.60	46.16 ± 4.43	0.021 p1 = 0.008 p2 = 0.01 p3 = 1
DCP-VD Whole Image (%)	45.96 ± 5.11	43.74 ± 4.61	44.91 ± 4.86	0.013 p1 = 0.007 p2 = 0.381 p3 = 0.480
DCP-VD Superior Hemi (%)	46.33 ± 4.99	43.78 ± 4.85	45.14 ± 5.00	0.005 p1 = 0.002 p2 = 0.346 p3 = 0.372
DCP-VD Inferior Hemi (%)	45.58 ± 5.41	43.66 ± 4.80	44.69 ± 5.15	0.041 p1 = 0.039 p2 = 0.568 p3 = 0.650
DCP-VD Fovea (%)	29.94 ± 7.07	27.62 ± 8.06	29.93 ± 7.81	0.002 p1 = 0.002 p2 = 1 p3=0.001
DCP-VD Parafovea (%)	48.99 ± 4.87	47.37 ± 5.29	47.81 ± 4.81	0.05 p1 = 0.037 p2 = 0.155 p3 = 1
DCP-VD Perifovea (%)	47.00 ± 5.69	44.86 ± 4.79	45.92 ± 5.48	0.034 p1 = 0.02 p2 = 0.503 p3 = 0.722
FAZ-A (Foveal Avascular Zone Area, mm²)	0.42 ± 0.29	0.42 ± 0.29	0.42 ± 0.30	> 0.05
FAZ-P (FAZ Perimeter, mm)	2.68 ± 1.59	2.73 ± 1.58	2.76 ± 1.88	0.131
FD-300 (%)	50.18 ± 4.22	48.75 ± 5.65	48.78 ± 4.18	0.005 p1 = 0.008 p2 = 0.013 p3 = 1
RPCP-VD SV Whole Image (%)	46.56 ± 3.77	46.18 ± 3.26	46.21 ± 3.67	0.150
RPCP-VD SV In-disc (%)	49.21 ± 6.36	48.68 ± 6.43	49.07 ± 5.60	0.241
RPCP-VD SV Peripapillary (%)	48.20 ± 4.47	48.09 ± 4.43	47.60 ± 4.74	0.068
RPCP-VD AV Whole Image (%)	53.79 ± 3.82	52.96 ± 3.85	53.33 ± 3.74	0.091
RPCP-VD AV In-disc (%)	58.98 ± 5.55	57.85 ± 5.60	58.93 ± 4.94	0.014 p1 = 0.02 p2 = 0.938 p3 = 0.176
RPCP-VD AV Peripapillary (%)	55.31 ± 4.37	54.97 ± 4.33	54.54 ± 4.65	0.024 p1 = 0.203 p2 = 0.029 p3 = 0.966

p*: Repeated measures ANOVA or Friedman test overall *p*-value

p1: Comparison between pre-PRP and 30 min after PRP

p2: Comparison between 30 min and 1 month after PRP

p3: Comparison between pre-PRP and 1 month after PRP

*PRP* Panretinal Photocoagulation, *CMT* Central Macular Thickness, *SFCT* Subfoveal Choroidal Thickness, *SCP* Superficial Capillary Plexus, *DCP* Deep Capillary Plexus, *VD* Vessel Density, *FAZ-A* Foveal Avascular Zone Area, *FAZ-P* FAZ Perimeter, *FD-300* Vessel density within a 300 μm-wide ring around the FAZ, *RPCP* Radial Peripapillary Capillary Plexus, *SV* Small Vessels, *AV* All Vessels

No significant changes were observed in CMT immediately after PRP (*p* > 0.05); however, a significant increase was noted at the one-month follow-up (*p* < 0.001). SFCT showed a nonsignificant increase at one month (*p* = 0.084).

**Fig. 2:**
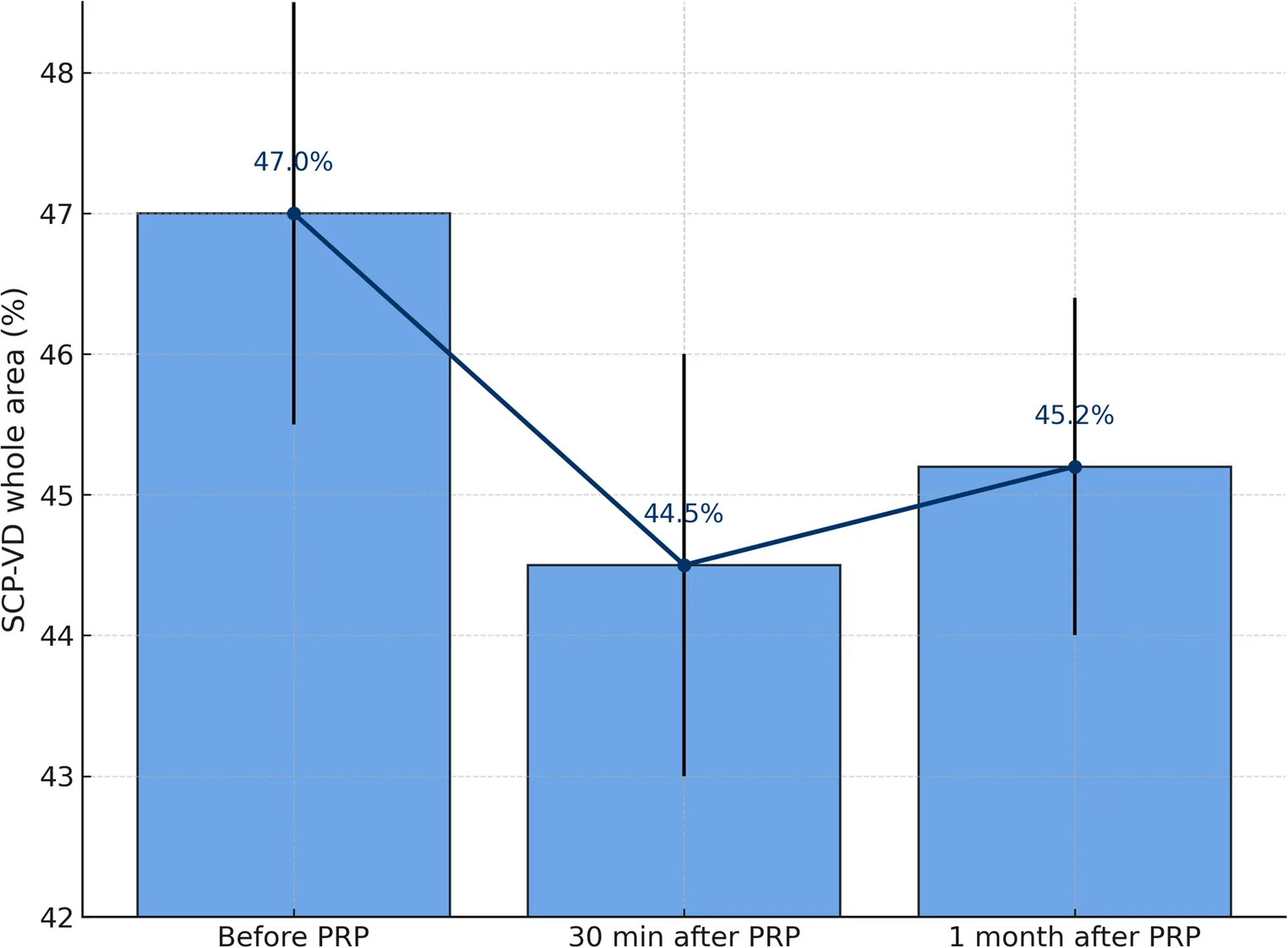
Changes in superficial capillary plexus vessel density (SCP-VD) in the whole image area measured at baseline, 30 min, and 1 month after panretinal photocoagulation (PRP)

SCP-VD in the fovea did not change significantly after PRP (*p* = 0.319); however, significant reductions were observed 30 min post-PRP in SCP-VD for the whole image, parafovea, perifovea, superior hemi, and inferior hemi (*p* = 0.002, 0.0006, 0.008, 0.006, and 0.012, respectively). Although these values partially recovered by the one-month follow-up, they remained significantly lower than baseline in selected regions (*p* = 0.01, 0.02, and 0.026, respectively), (Fig. 2).

**Fig. 3:**
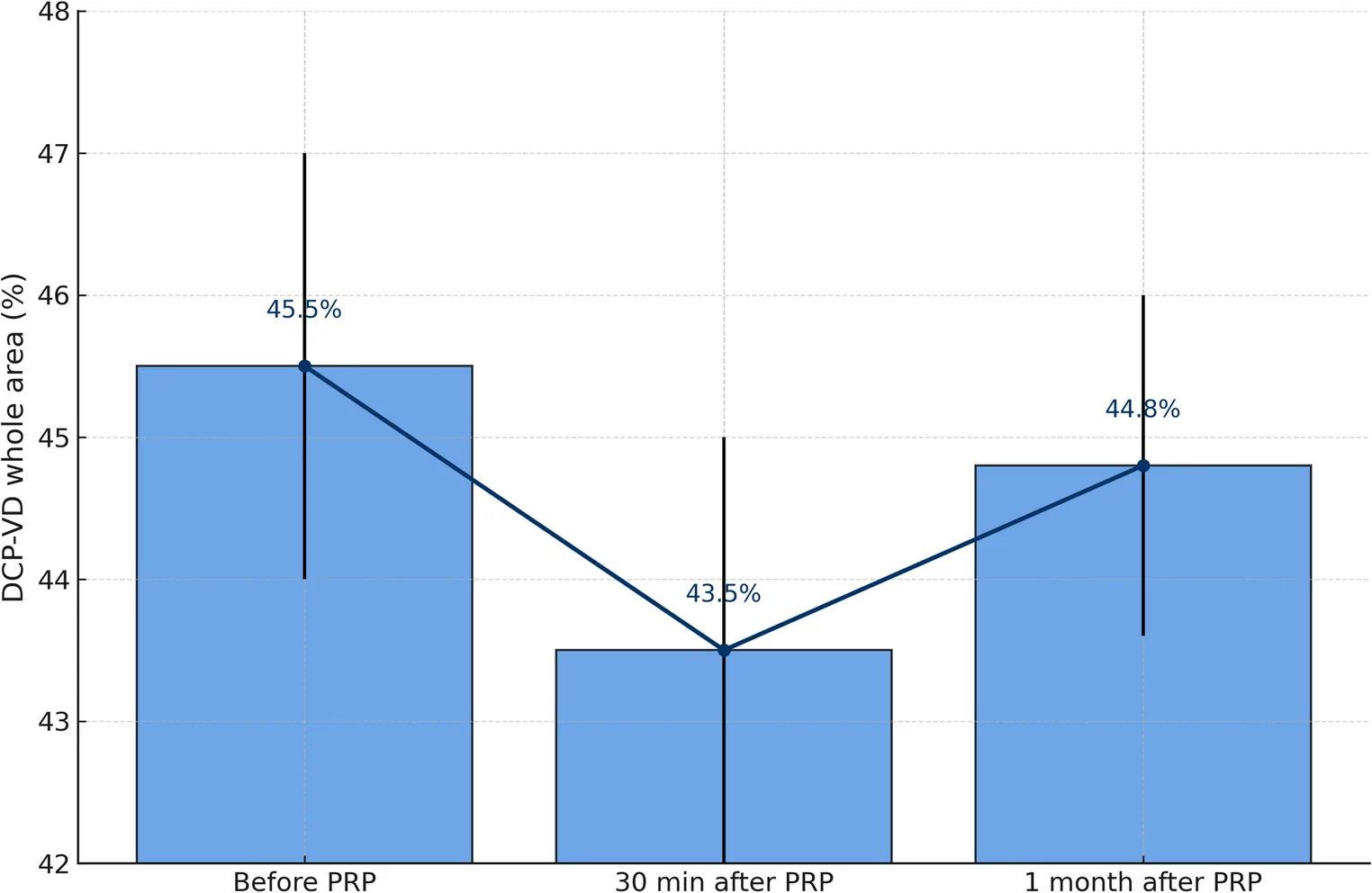
Longitudinal changes in deep capillary plexus vessel density (DCP-VD) in the whole image area before and after PRP

**Fig. 4:**
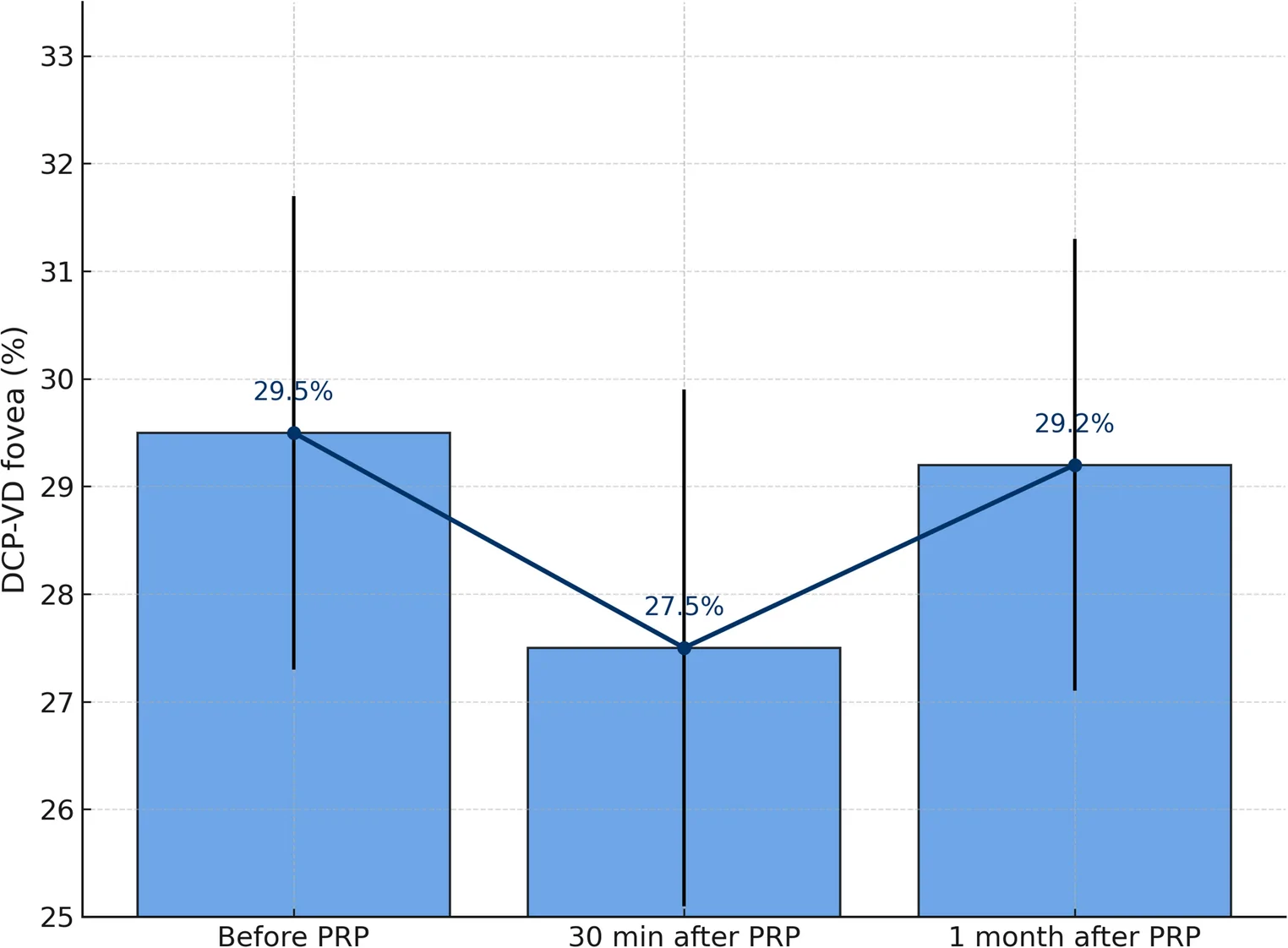
Changes in foveal deep capillary plexus vessel density (DCP-VD) at baseline, 30 min, and 1 month following PRP

For DCP-VD, significant decreases at 30 min post-PRP were observed for the whole image, superior hemi, and inferior hemi (*p* = 0.007, 0.002, and 0.039, respectively). By one month, these parameters had returned to baseline levels (*p* > 0.05). DCP-VD in the fovea was significantly reduced 30 min post-PRP compared to both baseline and one-month values (*p* < 0.001 for both), with no difference between baseline and one month (*p* = 0.604). DCP-VD in the parafovea and perifovea similarly decreased 30 min post-PRP (*p* = 0.037 and 0.02, respectively) but returned to baseline by the one-month follow-up (*p* > 0.05) (Figs. 3 and 4).

**Fig. 5:**
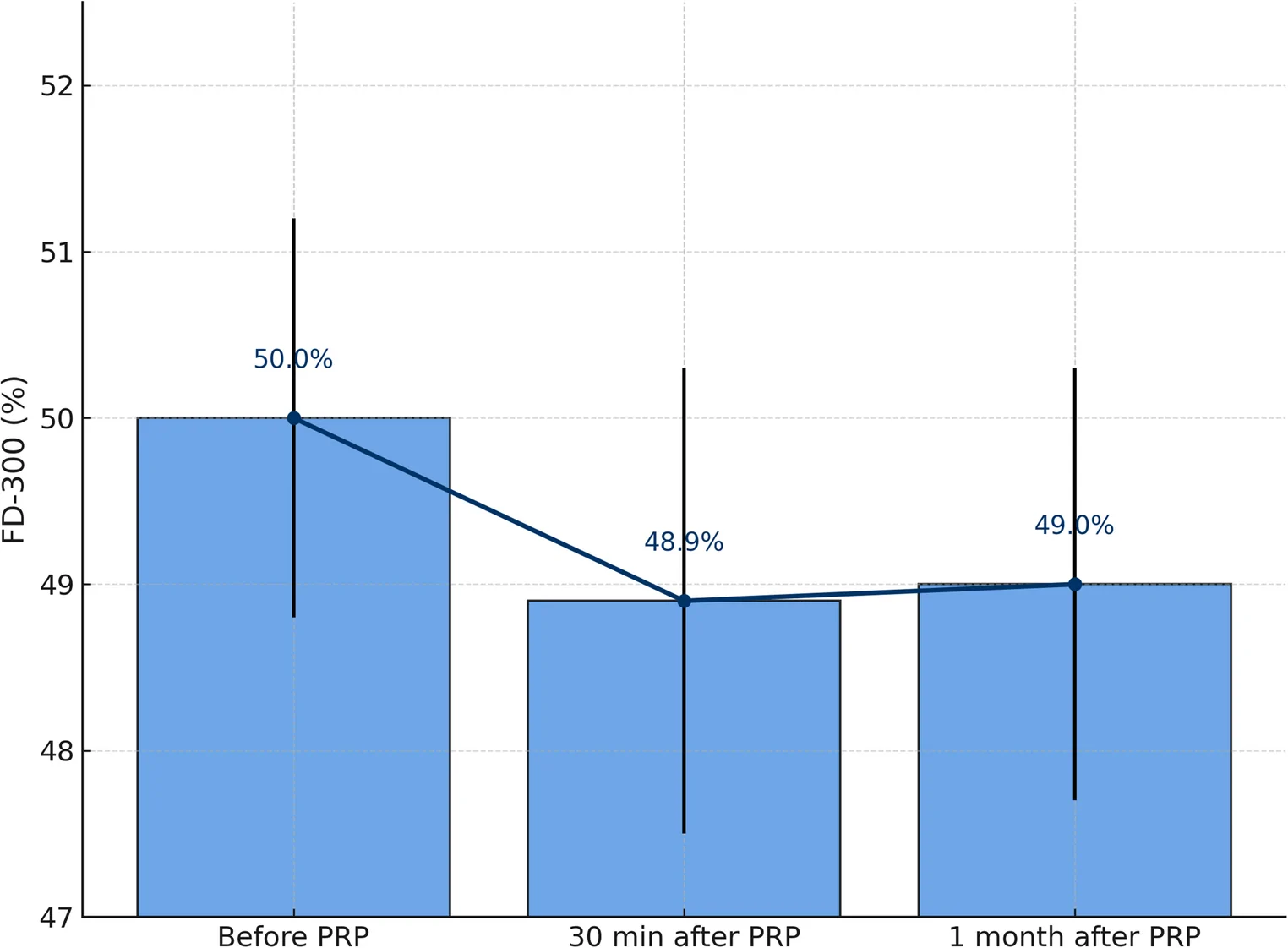
Alterations in FD-300 measurements before and after PRP

No significant differences were observed in FAZ-A or PERIM (*p* > 0.05). FD-300 (%) measurements were significantly lower both 30 min post-PRP and at one month compared to baseline (*p* = 0.008 and 0.013, respectively) (Fig. 5).

**Fig. 6:**
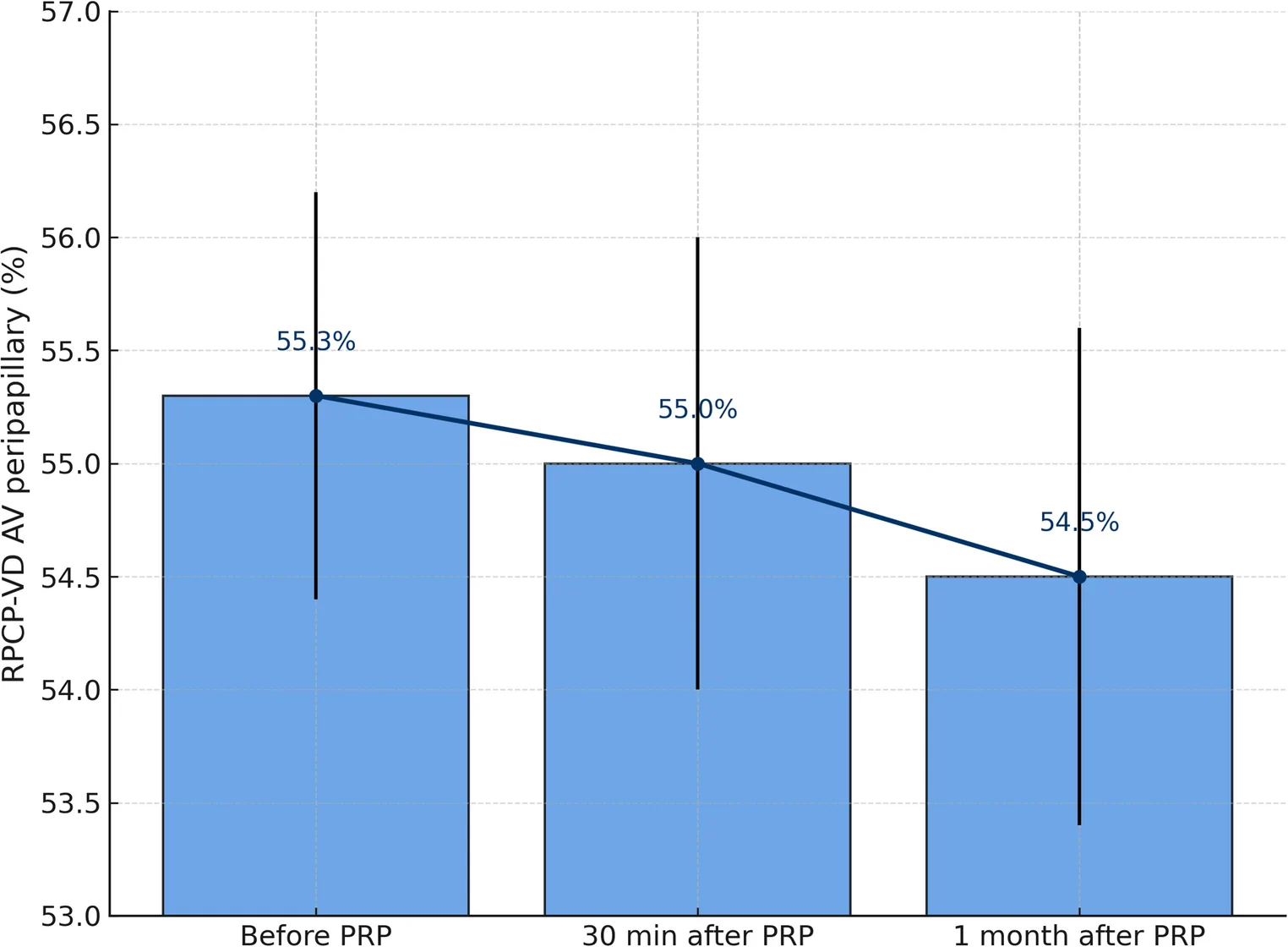
Changes in radial peripapillary capillary plexus vessel density (RPCP-VD) following PRP

Among patients without NVD, RPCP-VD-SV showed no significant changes in the whole image, inside disc, or peripapillary regions (*p* = 0.150, 0.241, and 0.068, respectively). RPCP-VD-AV showed no significant change in the whole image (*p* = 0.091); however, inside disc values decreased significantly 30 min post-PRP (*p* = 0.020), while peripapillary values decreased significantly both 30 min post-PRP and at the one-month follow-up (*p* = 0.029) (Fig. 6).

*P*-values for longitudinal comparisons were displayed directly on the figures to facilitate visualization of the acute (30-minute) and subacute (1-month) vascular effects of PRP.

The mean laser power used during PRP was 1658.48 ± 312.22 mW, with an average of 3165.45 ± 610.67 laser spots and a total LAA of 382.03 ± 73.70 mm². Correlation analyses between LAA and changes in OCTA macular and optic disc parameters at the one-month follow-up revealed no significant associations (*r* < 0.3 for all parameters).

## Discussion

In this prospective OCTA-based study, we identified distinct temporal microvascular responses following panretinal photocoagulation in eyes with advanced diabetic retinopathy. The principal findings were: (1) a significant acute reduction in both superficial and deep capillary plexus vessel density at 30 min; (2) partial recovery of DCP parameters by one month, whereas selected SCP measurements remained persistently reduced; (3) a sustained decrease in FD-300 despite stable FAZ area and perimeter; (4) significant reductions in peripapillary RPCP-VD, particularly in arterio-venous measurements; and (5) the absence of correlation between total laser ablation area and OCTA parameter changes. Together, these findings suggest that PRP induces an immediate hemodynamic response followed by differential subacute vascular adaptation across retinal plexuses. The relatively high mean HbA1c level in our study population indicates poor glycemic control and represents an important factor when interpreting retinal vascular changes. While the acute OCTA alterations observed at 30 min after PRP are unlikely to be explained by the natural course of diabetes alone, the vascular changes detected at the one-month follow-up may reflect a combination of laser-induced effects and ongoing microvascular dysfunction associated with uncontrolled diabetes. Therefore, attribution of subacute vascular changes solely to PRP should be made with caution.

The inclusion of a 30-minute evaluation provides insight into a hyperacute phase of vascular response that has been largely overlooked in prior OCTA studies. The significant reduction in both SCP and DCP vessel density at this early time point suggests that PRP induces an immediate hemodynamic disturbance rather than delayed structural capillary dropout. This observation may help reconcile inconsistencies in the literature, as studies evaluating vascular parameters only at one month or later may be capturing a partially stabilized or adaptive phase rather than the initial response. By documenting this early reduction, our findings highlight that PRP-related microvascular alterations begin within minutes and may not necessarily reflect permanent vascular damage.Laser photocoagulation produces controlled thermal injury, which may lead to transient vasoconstriction, cytokine release, and redistribution of retinal blood flow.

The differential recovery patterns observed between the superficial and deep capillary plexuses may reflect fundamental differences in their anatomical organization and physiological roles. The deep capillary plexus is closely associated with the outer retinal layers and is thought to play a critical role in meeting the metabolic demands of the photoreceptors. This close coupling may confer a more dynamic autoregulatory capacity, allowing the DCP to rapidly adapt to transient hemodynamic or inflammatory disturbances induced by PRP. Furthermore, inflammatory mediators released after PRP may exert a more prolonged effect on the SCP, whereas the DCP may be more susceptible to transient perfusion redistribution with subsequent recovery. Consistent with this concept, previous OCTA-based anatomical studies have demonstrated that the DCP exhibits a highly interconnected vascular architecture closely linked to outer retinal metabolic regulation, potentially facilitating faster functional recovery after transient insults [11]. Together, these factors may explain why DCP vessel density returned to baseline by one month, while selected SCP parameters remained persistently reduced.

Experimental and clinical studies have shown that PRP induces an acute inflammatory response characterized by rapid upregulation of cytokines and growth factors. Previous studies measuring intraocular cytokine levels have demonstrated significant increases in vascular endothelial growth factor (VEGF) and interleukin-6 (IL-6) shortly after retinal photocoagulation [12, 13]. These mediators are known to influence vascular tone, permeability, and autoregulatory mechanisms, and their early release may contribute to transient vasoconstriction and redistribution of retinal blood flow. In this context, the significant reduction in vessel density observed at 30 min in our study may reflect a cytokine-mediated hemodynamic response rather than immediate structural capillary loss, providing biological plausibility for the ultra-early OCTA findings.

Our findings both align with and differ from previous OCTA studies evaluating PRP-induced vascular changes. Kim et al. reported a reduction in both SCP and DCP vessel density at one month, followed by partial recovery at later time points [8]. Similarly, Faghihi et al. observed decreased macular perfusion shortly after PRP [6]. In contrast, Lorusso et al. found no significant change in macular perfusion parameters following PRP with shorter pulse durations [14]. Notably, Abdelhalim et al. reported significant increases in SCP and DCP vessel density across the whole image, foveal, and parafoveal regions at one and six months after PRP in 30 eyes of 18 patients with proliferative diabetic retinopathy [15]. The variability across studies may relate to differences in laser parameters, patient characteristics, metabolic control, and—importantly—the timing of OCTA evaluation. By incorporating an ultra-early 30-minute assessment, our study demonstrates that an immediate vascular reduction may precede either stabilization or recovery, potentially explaining discrepancies in previously published results.

Interestingly, FAZ area and perimeter remained stable following PRP in our cohort, whereas FD-300 showed a significant and sustained decrease. Previous OCTA studies have reported inconsistent FAZ responses after PRP; while some authors have described FAZ reduction at follow-up, others, including Mirshahi et al. and Zhao et al., reported no significant FAZ changes after treatment [16, 17]. In line with these latter findings, FAZ metrics remained unchanged in our study despite measurable alterations in parafoveal perfusion. Because FD-300 reflects capillary density in the parafoveal region independent of FAZ size, this dissociation suggests that subtle microvascular perfusion changes may occur without measurable FAZ expansion. This observation highlights the potential value of FD-300 as a more sensitive biomarker of early macular vascular alteration following PRP.

The analysis of radial peripapillary capillary plexus parameters revealed significant reductions, particularly in arterio-venous measurements, with peripapillary changes persisting at one month. The peripapillary microvasculature runs parallel to the retinal nerve fiber layer and may be particularly vulnerable to both diabetic microangiopathy and PRP-induced hemodynamic redistribution. Notably, these alterations occurred independently of total laser ablation area. This suggests that optic nerve head microvascular responses may be influenced more by biological vascular regulation and systemic metabolic status than by the sheer extent of peripheral laser application.

Peripapillary vascular alterations have been documented in diabetic retinopathy independent of PRP. Vujosevic et al. demonstrated reduced RPCP-VD correlating with neural damage even before overt clinical signs, while Greig et al. suggested that peripapillary OCTA metrics may predict disease progression [18, 19]. Although Zhao et al. did not observe significant RPCP changes after PRP [17], our findings suggest that subtle peripapillary alterations may occur early and persist in the subacute phase, potentially influenced by imaging timing and patient characteristics.

Despite applying a substantial number of laser spots and calculating total laser ablation area, no significant correlation was found between LAA and OCTA parameter changes. According to the Diabetic Retinopathy Study (DRS), effective panretinal photocoagulation typically requires at least 1500 single-spot laser applications [20]. In the present study, the mean laser power and the number of laser spots were higher than those reported in earlier conventional PRP protocols. This difference can be attributed to the use of pattern scan laser technology with short pulse durations, which produces smaller retinal burns and therefore necessitates a greater number of laser spots to achieve adequate therapeutic coverage. Importantly, despite the higher spot counts, no significant correlation was observed between total laser ablation area and changes in OCTA-derived vascular parameters.

Taken together, our results suggest that early OCTA changes after PRP should be interpreted cautiously, as acute reductions in vessel density may reflect transient hemodynamic responses rather than true disease progression. The partial normalization observed in the deep capillary plexus further supports the presence of vascular adaptation over time, underscoring the importance of imaging timing when interpreting post-PRP OCTA findings in both clinical practice and research settings.

The strengths of this study include its prospective design, the combined evaluation of macular and optic nerve head microvasculature, the assessment of ultra-early vascular responses, and the quantitative calculation of total laser ablation area. Nevertheless, several limitations must be acknowledged. The follow-up period was relatively short, limiting assessment of long-term vascular remodeling. The absence of a control group and the generally poor systemic metabolic control of the cohort may have influenced vascular measurements. Additionally, OCTA is subject to segmentation errors and projection artifacts, and we did not evaluate other vascular features such as microaneurysms or IRMA. Future longitudinal studies with larger cohorts and extended follow-up are needed to better characterize the long-term microvascular consequences of PRP.

Importantly, the novelty of this study lies in its temporal approach to PRP-related microvascular changes. By integrating ultra-early (30-minute) and subacute (one-month) OCTA assessments and evaluating both macular and optic nerve head microvasculature, our study provides a comprehensive characterization of PRP-induced vascular dynamics. These findings suggest that early OCTA changes predominantly reflect transient hemodynamic responses rather than irreversible capillary loss, highlighting the clinical importance of imaging timing when interpreting post-PRP OCTA findings.

In conclusion, PRP induces measurable and temporally dynamic microvascular alterations in both the macula and optic nerve head. The inclusion of an ultra-early (30-minute) assessment demonstrates that these changes begin within minutes and likely represent an acute hemodynamic response rather than immediate structural capillary loss. The subsequent layer-specific adaptation observed at one month further supports the dynamic nature of PRP-induced vascular regulation.

## Data Availability

The data that support the findings of this study are not publicly available but are available from corresponding author (SD).
